# A New Perspective for the Future of Male Infertility Treatment and Research

**Published:** 2020

**Authors:** Mohammad Reza Sadeghi

Fertility and childbirth have been one of the basic needs throughout the human life, which in some cases are failed despite the couple’s long efforts. Infertility is defined as the disability of childbearing following one year of unprotected intercourse. Infertility has a prevalence of 10–15% among couples at the reproductive age and therefore it is one of the almost common illnesses for individuals. According to evidence based clinical data, the female and male partners’ role in causes of infertility are approximately equivalent. But the male’s and female’s role in infertility has been unrealistically underestimated or overemphasized in different communities. In fact, the common practice is ignoring the men’s role in the causes of infertility and to highlight the role of women in infertility. But over the time and advancement of infertility diagnosis and treatment, the insights into the causes and role of male and female in infertility have become more realistic (1). A quick review on number of researches in each area of male factor and female factor infertility over the past 50 years is also evidence of this fact.

**Figure:**
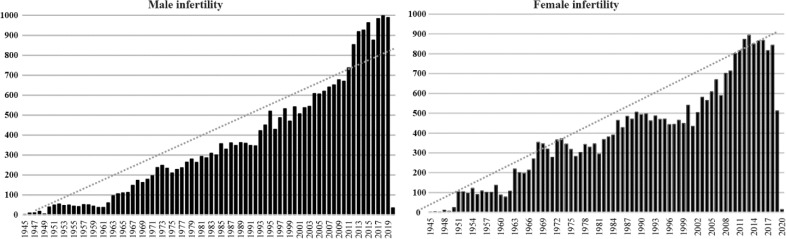
Quick search on Pubmed database with keywords “Male Infertility” and “Female Infertility” in the period 1945–2019

Although the researches on male and female infertility are increasing with equal rate, but recently, most interventions, surgeries and assisted reproductive technologies (ARTs) for infertility treatments are performed on women, even in cases of a normal female with male factor infertility. However, introduction of Intrauterine Insemination (IUI), In Vitro Fertilization (IVF), Intracytoplasmic Sperm Injection (ICSI), *Testicular Sperm* Aspiration (TESA), Percutaneous Epididymal Sperm Aspiration (PESA), Testicular Sperm Extraction (TESE), and Microdissection Testicular Sperm Extraction (Micro-TESE) have revolutionized treatment of infertility, especially in patients with a male factor. However, in all of these treatments, a woman who is fertile should be super-ovulated and ovum pick-up under anesthesia needs to be done and followed by a wide range of hormonal and non-hormonal medications to prepare the uterus for embryo transfer. Taking these hormones will continue for several months following successful pregnancy which causes a lot of suffering and pain for the woman, and this situation will be much worse if ovarian hyperstimulation syndrome (OHSS) or multiple pregnancy occurs. Another problem is the low success rate of ART cycles which will require repeating treatment cycles that may result in more physical and psychological damages for women (2).

Therefore, isn’t it necessary to seek new therapies for male factor infertility that do not harm women? There is so many researches currently underway to increase the success rate of ART cycles. Introducing and applying different technologies such as Time-lapse Incubators (TL) and Preimplantation Genetic Testing for Aneuploidies (PGT-A) have improved selection of best embryos, which in part leads to increased success of IVF cycles and reduced rate of multi-pregnancy (3). In addition to the above technologies, natural IVF cycle was introduced to reduce the harm in women, but it also needs regular sonographic monitoring of ovary, ovulation triggering, surgical ovum pick-up, *in vitro* fertilization, embryo culture and also low success rate of natural cycle is similar to routine IVF cycles (2). Therefore, it seems that all efforts to minimize the harm and inconvenience for women and increasing success rate of IVF cycles have not been successful so far.

Therefore, in such conditions, serious changes in direction of diagnostic methods and treatment procedures of male infertility are more crucial than ever. Though it is conceivable that future research on male infertility will lead to the development of new approaches to infertility treatment that no longer require multiple medications and surgery on women with nearly 100% success rate, the idea is so far-fetched and fanciful at present and is more like a science fiction. But historical experience has shown that everything that has once been human imagination and legend has become reality over time and is now available for us as common and routine technologies. Therefore, whatever we conceive as a way to improve infertility treatments with a high success rate without harm to women will certainly be achieved with efforts of scientists and support of clinicians in future.

## References

[1] Inhorn MC Patrizio P. Infertility around the globe: new thinking on gender, reproductive technologies and global movements in the 21st century. Hum Reprod Update. 2015;21(4):411–26. 25801630 10.1093/humupd/dmv016

[2] Niederberger C Pellicer A Cohen J Gardner DK Palermo GD O’Neill CL Forty years of IVF. Fertil Steril. 2018; 110(2):185–324.e5. 30053940 10.1016/j.fertnstert.2018.06.005

[3] Reignier A Lammers J Barriere P Freour T. Can time-lapse parameters predict embryo ploidy? a systematic review. Re-prod Biomed Online. 2018;36(4):380–7. 10.1016/j.rbmo.2018.01.001 29398421

